# A survey of direct-to-consumer genotype data, and quality control tool (*GenomePrep*) for research

**DOI:** 10.1016/j.csbj.2021.06.040

**Published:** 2021-06-27

**Authors:** Chang Lu, Bastian Greshake Tzovaras, Julian Gough

**Affiliations:** a MRC Laboratory of Molecular Biology, Francis Crick Avenue, Cambridge Biomedical Campus, Cambridge, UK; b Center for Research and Interdisciplinarity (CRI), Universite de Paris, INSERM U1284, Paris, France

**Keywords:** Genotyping, Direct-to-consumer sequencing, Open genome, Personal genome, SNP arrays

## Abstract

Two major forces have contributed to the fast growth of human genetic data. One from medical research supported by governments and academic institutes; the other from direct-to-consumer (DTC) sequencing companies. While the former benefits from meticulously designed sequencing standards and quality control procedures, the latter comes in various formats and sequencing methods which are subject to changes over time and the particular needs of different companies. Thanks to the general public who shared their DNA data without constraint, here we provide a review for over 7000 genomes made public between 2011 and 2020, and produced by over six DTC sequencing companies. An open source tool-kit to systematically parse, quality check and filter genome files and statistically problematic alleles is provided to prepare consumer DNA datasets for research. The *GenomePrep* output is available in two common DNA datafile formats to enable further analysis with other tools. We also provide for download the combined output for all OpenSNP array genomes processed in this paper in a single data freeze file.

## Highlights

• Review of the offerings from commercial genotyping companies.
• Analysis of consumer genotype data SNP arrays.
• Quality control analysis of over 7000 open genomes.
• Open source tools and web service providing quality control report of genotype arrays.

## Graphical abstract

**Figure:**
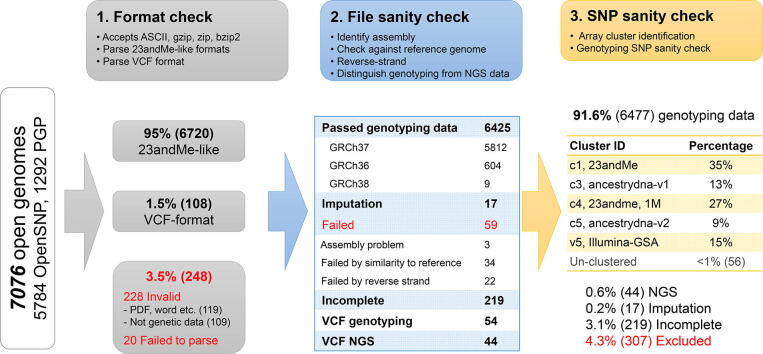

## 1 Introduction

Customers of the direct-to-consumer (DTC) genotyping companies represent the majority of the population who have had their genome read. This group is under-exploited by the academic genetics community for research. At the time of writing in Jan 2021, the two largest sequencing companies AncestryDNA and 23andMe officially report 30 million customers in total [1], [2], and 1.4 million and 1 million in research cohort size respectively for the Global Alliance for Genomics & Health (GA4GH), the international consortium for sharing genomic data [3]. These are people who have paid for sequencing, have ownership of their personal raw DNA data and the freedom to choose how they wish to use it [4]. Many of these consumers have shown interest in understanding their genomes better by taking the initiative in paying for third-party services like Xcode Life, CodeGen etc. These people represent a huge potential group of participants for genetic research that is seeking to validate findings by a random or untargeted cohort, or trying to study the genetics for a particular phenotype under limited budget.

One of the main hurdles in utilizing consumer DNA data for research is that these data vary greatly in sequencing methods and, most importantly, the data quality. For the majority of consumers who have had their DNA genotyped, the versions of genotyping microarray differ for different companies at different time periods. The sequencing methods are often selected and designed based on the individual marketing needs of the companies and the DNA data are processed by a different bioinformatics team in each company. Researchers will not have access to the internal quality control data on the samples/arrays from the various companies; they most often have to deal with whatever raw download format the DNA data are provided to the customer in, which may have undergone varying amounts or types of quality control.

These limitations mean that mass genetic data are most useful to the DTC company responsible for the genotyping, or partners who have commissioned a large project. Here, we will firstly provide a review of the commercial providers, their offering of sequencing methods and genotyping arrays. Secondly, over 7000 genomes were downloaded from open genetic data sharing platforms, the majority coming from the OpenSNP project [5]. The genomes were parsed, processed and analysed for comparison and to create a pipeline for specific quality control of consumer genomes, thus enabling general genetics research on these data. We have made open source code, a web interface available for user to upload and run the pipeline, and a data-freeze of over 5000 OpenSNP genomes post-process.

## 2 Review of genotyping arrays commercial providers

Genotyping refers to the usage of single nucleotide polymorphism (SNP) arrays to genotype human DNA at ten-thousands to a million SNPs across the genome simultaneously [6], [7]. Because it can effectively gather information on SNPs of known importance at a relatively low cost, genotyping remains the most common choice for consumer genetic data sequencing.

Late 2000 to early 2010 marked significant technological development of genotyping arrays, during which time 2 methods from Affymetrix and Illumina, namely the Affymetrix GeneChip (SNP5.0, SNP6.0) and Illumina BeadChip (HumanHap550 and Omni family), stood out. These have been extensively reviewed on their technology and historical development [6], [7], genotype calling software [8], and genome coverage evaluation for GWAS studies [9]. Both the Affymetrix and the Illumina methods for SNP genotyping have been widely used, and reported to achieve above 99.5% accuracy in genotype calling [7], [8]. With accuracy levels competitive to each other, the two companies designed their services to cater for different sequencing and cost-effectiveness needs of customers.

Illumina has occupied most of the consumer genomics market. Almost all of the top DTC companies are using the Illumina genotyping arrays (Table 1). The consumer sequencing giant 23andMe have changed its choice of genotyping array 5 times since the company founded in 2006, but remained closely tied with Illumina. Three companies that have focused on ancestry detection, namely AncestryDNA, FamilyTree DNA and My Heritage, have mostly retained their choice with the Illumina OmniExpress chip, although each have different custom selected or excluded SNPs of their own specific design. To design the current Global Screening Array chip, Illumina started a consortium and collaborated with research institutions as well as DTC companies.

**Table 1:** List of genotyping arrays commonly used by DTC companies.

Chip ID	Size	Rationale & reference	DTC companies*
Illumina HumanHap550	~560,000	Derived from the International HapMap project, mostly targets 4 populations in the project: Caucasian, Han Chinese/Japanese and Yoruba [13]	23andMe v1, v2 deCODEme
Illumina OmniExpress	~730,000 +/− selected	Derived from the 1000 Genomes project, designed to target a specific MAF range. OmniExpress cover common variants down to 5% MAF [14]	23andMe v3 AncestryDNA FamilyTree DNA MyHeritage
Illumina HTS iSelect (HD)	Custom select	More cost effective than OmniExpress with a smaller set of SNPs [15]	23andMe v4
Illumina GSA	~650,000	Designed to have better imputation accuracy, to cover variants from ClinVar and special markers, and to achieve high-value for cost [16]	23andMe v5 Living DNA FamilyTree DNA (2019)
Illumina CoreExome	~570,000 + selected	Customisable chip offering economical way to perform large genetic studies. Developed in collaboration with research institutions [17]	Genes for good (academic study, non-commercial) [18]
Illumina ASA	~650,000	The GSA Asia chip included Asian-specific markers	WeGene (China) Diagnomics (South Korea)
Affymetrix (Thermo Fisher) UKBiobank Axiom Array	~820,000	Imputation-aware SNP selection, optimized for GWAS, low-frequency SNPs of European and British ancestry [10], [19]	Living DNA (2019)

* The list is not exhaustive.

Affymetrix (Thermo Fisher) has mostly worked with population genomics studies from research institutions. For instance, its custom UK-Biobank chip that genotyped 500,000 people [10].

The most appealing advantage of genotyping arrays over next generation sequencing (NGS) is the cost. Therefore, when designing a SNP array chip, it is always necessary to consider targets: whether to cover as many common SNPs as possible for an international audience, or to target a specific population with more population-specific SNPs included, or to target a particular disease or phenotype by including relevant rare SNPs. Also because of the need for cost-effectiveness, the chip size is essentially capped at 1 million, with most products having 600 K to 800 K SNPs, which would represent less than 1% of the over 100 million validated human polymorphisms in dbSNP database [11].

The size limit of genotyping arrays has also meant they would be likely to have only a small overlap with each other. For example, two of Illumina’s most popular products, OmniExpress and GSA, have only 20% overlap by SNP positions, as shown by a LivingDNA blog post [12] and revealed by data analysis in this paper (Section 3.2). This represents a potential problem in cross comparison of genetic data produced by different types of genotyping arrays.

### 2.1 On direct-to-consumer Next-Generation sequencing (NGS)

Next-generation sequencing includes whole genome sequencing (WGS) and whole exome sequencing (WES). Instead of using chip arrays, which use short nucleotides to exact-match stretches of DNA with a predefined SNP, NGS sequencing will read longer overlapping stretches of DNA de novo and align them to the reference genome to see the differences [20]. With sufficient depth of overlap between reads, NGS will potentially find all variants in the genome, common or rare. A depth of e.g. 30x means that positions on the genome have been sequenced 30 times across individual overlapping reads.

Since the first human genome sequenced in 2013, the cost of whole genome sequencing has gone down significantly, reported to require minimum $1906 for whole genome sequencing (WGS) and $555 for whole exome sequencing (WES) at year 2018 [21], and is advertised at $800 for 30x WES in 2021 [22]. However, this is still over 10-fold the cost of genotyping, a price point that means NGS is still not the primary choice for the majority of consumers. There are not yet enough direct-to-consumer NGS data for the work in this paper, but it is the natural extension in the future when NGS inevitably replaces genotyping.

### 2.2 On imputation from SNP arrays

‘Imputation’ is commonly applied to genome arrays to give pseudo-coverage of the genome for positions that are not directly measured by the array [23]. These are statistically derived estimates using the principles of genomic inheritance. It should be noted that while reproducibility of current genotyping arrays is estimated at above 99.9% [14], [16], the accuracy of imputation depends on the type of SNP positions in the array, with even the Illumina GSA (among the best for imputation) lower than 91% for the easiest target of MAF > 5% [16]. Therefore, the most informative and trust-worthy data to use for research are those measured on the genotyping array, and secondly imputations with different levels of success rate for different version of arrays, then finally there are dark positions in areas of the genome that imputation doesn’t cover.

## 3 Analysis and comparison of genotyping arrays from consumer data

The above review of genotyping arrays (in Table 1) that are broadly provided by the sequencing companies relies on publications and media reports. As there is no universal access to the companies’ internal array data and pipelines, this is only of limited use in understanding the nature of the information contained in the raw DNA files they provide for download to their customers. Thus, we provide here an analysis of the arrays from a large sample of consumer data covering multiple companies. Thanks to the thousands of participants of the open genome projects who have donated their genetic data to the public good, the majority from the OpenSNP platform [24], we were able to identify various types of arrays used in consumer genotyping over the past decade by at least 6 different companies.

### 3.1 Public genomic data

In 2005, George Church talked about recruiting cohort participants who are consented based on expectation of full public data release [25], and initiated the Harvard Personal Genome Project (PGP) [26]. Independently in 2011, Bastian Greshake *et al.* set up the OpenSNP platform as a crowd-sourcing and crow-funded platform, for consumers of DTC companies to publicly share data and communicate with each other [5].

**Fig. 1:**
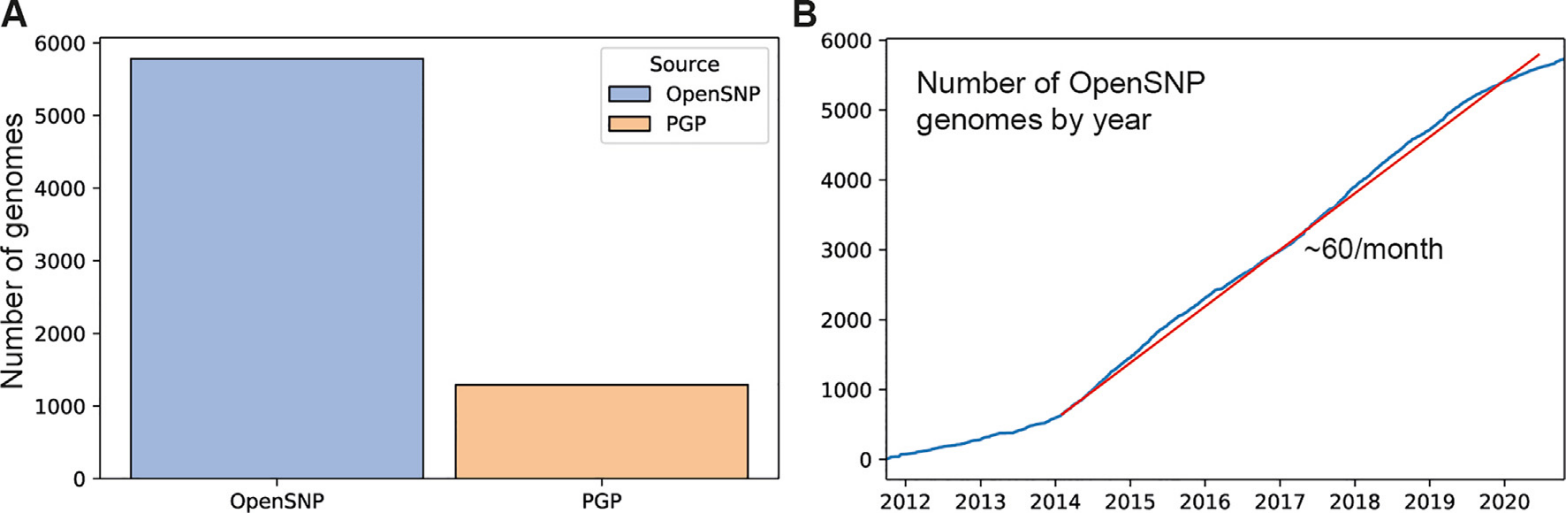
Overview of open genomes. (A) 7076 genetic data files were obtained from OpenSNP and the Harvard PGP cohort. (B) Accumulation of genomes on OpenSNP over the past 9 years. Since 2014, a steady rate of about 60 DNA data uploads per month is observed (red line). (For interpretation of the references to colour in this figure legend, the reader is referred to the web version of this article.)

For this work we used 4401 genomes from OpenSNP for SNP array comparison (Section 3.2) and 7076 from OpenSNP and PGP for quality control analysis (Section 4). A total of 5790 genomes were initially downloaded from the OpenSNP webserver in Oct 2020 [5]. We observed from the dates that a continuous supply of data must have accumulated over a period of 9 years (Fig. 1). At the time of writing, a few users have removed their data from the public domain, therefore we have restricted our analysis to the remaining 5784 genomes from 5258 users. By July 2020, the Harvard PGP server [27] hosted 3609 open genetic data files for 1375 participants. One third of the genetic data comes from Veritas Genetics, which is 1061 files for just 127 participants that contained large sequence alignment data (e.g. BAM) not yet processed with variant calling. These are excluded. Another one third of the data from PGP are duplicate files, secondary reports from a third party, and microbial genomes; these were all also excluded. In the end, manual data cleaning yielded 1292 plausible genetic data files for 1012 participants.

### 3.2 SNP array comparison

**Fig. 2:**
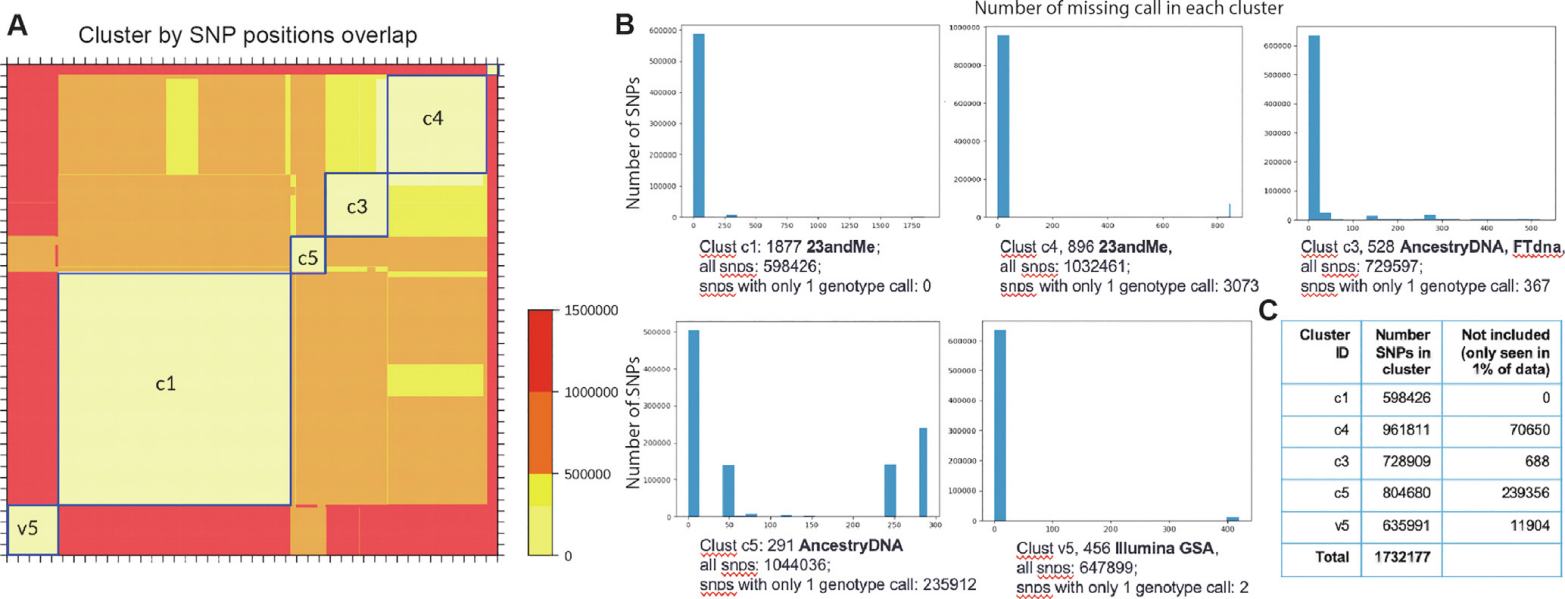
Clustering of genotype genetic data by similarity in the SNP coverage. (A) Heat-map of distance matrix. Distance is defined as the SNP positions not in common between a pair of files. The cutoff for clustering is set at 200 K. (B) Distribution of SNP by genotyping call coverage is plotted for each cluster. The bars on the left of each subplots show the number of SNPs that are common to 100% of files within the cluster. Bars on the right show the number of SNPs that are only seen in a small number of files within the cluster. (C) Table lists the exact number of SNPs in each cluster.

SNP arrays were analysed from 4401 OpenSNP open genomes downloaded at the time of analysis. This includes genetic data from 23andMe (~78%), AncestryDNA (~14%), Family tree DNA, My Heritage (8%) and small numbers from deCODEme, Genes-for-good (non-commercial), IYG, etc. Using OpenSNP data (method in Section 4.1), 5 major clusters of SNP arrays were identified (Fig. 2, Table 2).

**Table 2:** List of major clusters identified in Fig. 2.

Cluster ID	Company composition	Average SNPs	Chip base (deduced)	common in c1	common in c3	common in c4	common in c5	common in v5
c1	23andMe-v4	~600 K	HTS iSelect HD	–	43%	54%	47%	17%
c3	AncestryDNA-v1, Familytree DNA, My Heritage	~700 K	OmniExpress	50%	–	69%	64%	25%
c4	23andMe-v3	~960 K	OmniExpress plus	87%	94%	–	68%	28%
c5	AncestryDNA-v2	~670 K	OmniExpress plus	52%	60%	47%	–	23%
v5	23andMe-v5, Living DNA	~650 K	Illumina GSAs	18%	23%	19%	23%	–

The files investigated contain from 500 thousand to 1 million SNPs. The difference between two files is represented by ΔN, namely the number of SNPs not in common between the two files. It is observed that ΔN can be as large as 1.4 million, which means two data of size 500 thousand and 1 million only have 100 thousand SNPs in common with each other.

Using ΔN as the distance measure of SNP array difference between genotyping data, 5 major clusters of SNP arrays can be found after clustering (Fig. 2). The genotyping data in each cluster are more than 90% similarity with each other in the SNP array, although some files still contain positions that are not seen in 99% of other files (Fig. 2B).

The companies and possible chip array version for each cluster are deduced from both the clusters, and using header information in the data (Table 2). 23andMe data predominantly come from 3 types of arrays, which are deduced to be the Illumina HTS iSelect HD (c1), OmniExpress plus (c4) and GSA (v5). The c4 is the largest genotyping array of ~1 million positions, covering roughly 90% of the c1 and c3. The c3 is made of ancestry-targeted companies, including the Ancestry V1, FamilyTree DNA and My heritage. It is a reduced Illumina OmniExpress array that contains most of the SNPs useful for ancestry-detection. The second version of Ancestry chip array is also observed in the data as the cluster c5. Deduction of the above chip arrays were made with information taken from the International Society of Genetic Genealogy (ISOGG) wiki [28].

## 4 Genome processing and quality control pipeline

**Fig. 3:**
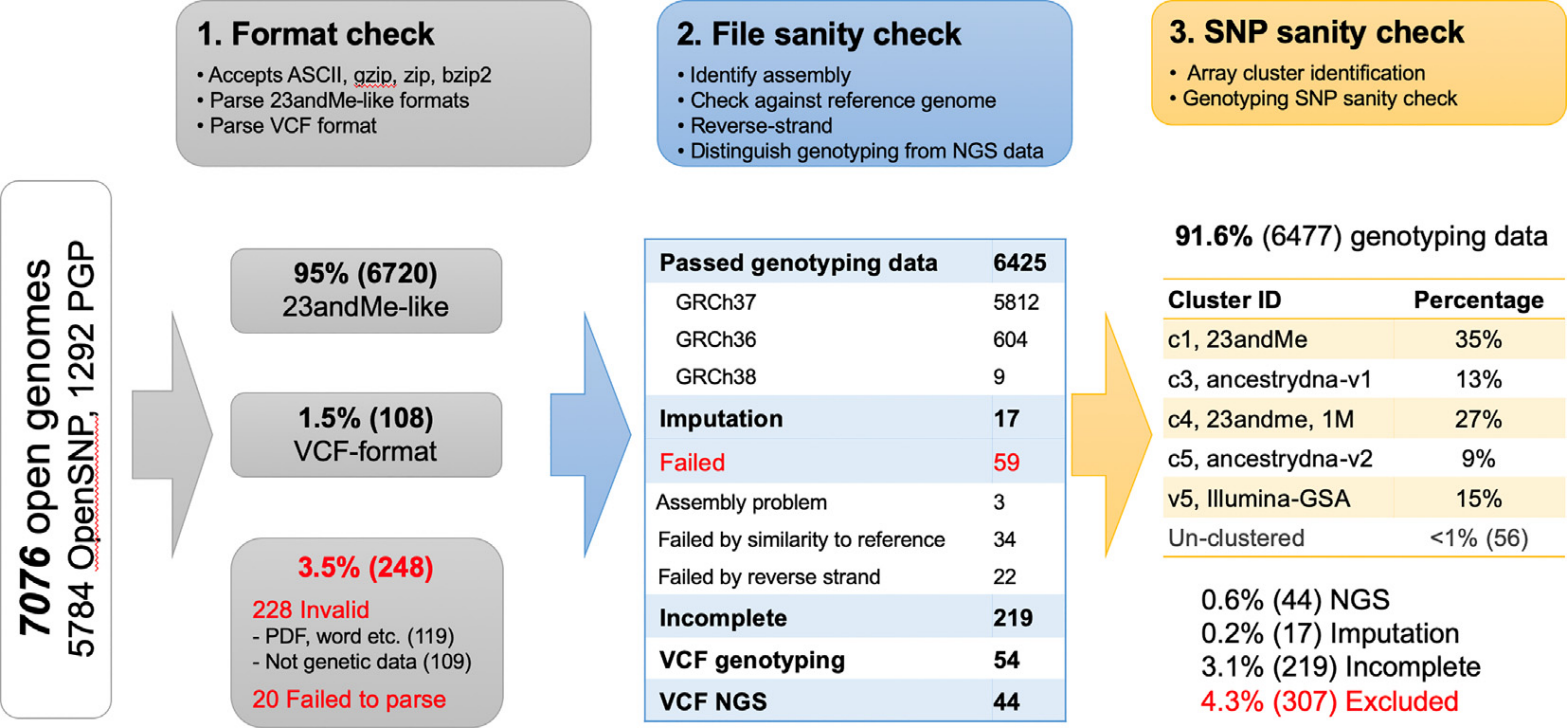
Summary flowchart of consumer genome processing pipeline. Three major steps of quality control are shown above and the respective results from processing 7076 open genomes are shown below, with corresponding colours. Red numbers indicate files that are excluded in various stages of the pipeline. In the end, less than 5% were excluded, and nearly 91% of the open genomes were found to be genotype data with a cluster assigned. These are subsequently subjected to SNP quality control, as per Section 4.3 and Fig. 4. (For interpretation of the references to colour in this figure legend, the reader is referred to the web version of this article.)

To demonstrate and facilitate the use of consumer data, we developed a quality control pipeline to systematically process them (Fig. 3). We make this pipeline available as ‘*GenomePrep’* via a web service (http://supfam.mrc-lmb.cam.ac.uk/GenomePrep/) and as an open source package on GitHub. Briefly, the pipeline includes parsing files of various formats from different companies, sanity checking the files and excluding ones that may have problematic data, then sanity checking the SNPs to exclude those with a statistical profile that is not credible. This section describes the details of the quality control procedure and results of processing over 7000 open genetic data files using the pipeline.

### 4.1 Input file format

Unlike research projects where data collection is organized and obtained from sequencing at source, consumer data collection is almost impossible to do consistently or reliably. There are Microsoft Word documents, files that have been edited, or files that are somehow rendered unusable by the consumer in the process of handling it.

In this procedure, PDF and word documents are excluded whereas text files are sent to the parser. Compressed files such as zipped, gzip, or bzip2 files are decompressed and included if found to contain one single genome flat file, but still excluded if the program can’t identify the genome file. For the genotyping files, the parser recognizes data files from companies that are included in the open genome dataset, e.g. 23andMe, AncestryDNA, Family Tree DNA, My Heritage, deCODEme etc. Due to some file formats being common or compatible between companies, the parser will likely work for some companies not explicitly listed. VCF-format files are also recognized and parsed.

### 4.2 File sanity check

While it is straightforward to identify and exclude unreadable files, it is more difficult to identify those that have undergone some level of corruption or edits, whether they intentional or unintentional alterations. The part of the procedure described below identifies important information about the genome, e.g. assembly, and sanity checks the data for: completeness; reasonable variations from reference genome; and to confirm the SNP’s calling strand is processed correctly. Suspicious data is excluded with a reason provided in the output.

#### 4.2.1 Identify the assembly version and compare to the reference genome

In the first step, the assembly is determined from the genetic file through header lines and a few known signature SNPs, defaulting to GRCh37 if the above information is not found. Then using this version of the assembly, the genetic files are compared to the respective reference genome to check whether the data presents a reasonable human genome with a plausible number of variants. Option is provided to convert genetic data to different versions of genome assemblies through CrossMap [29].

#### 4.2.2 Check the reverse strand SNP calling

It is a known problem in the field that SNPs are not always reported consistently on the same strand for all companies. This information is usually missing from the final data. In addition, there is no access to the companies’ internal quality check procedure on the samples, only the final results. Therefore we attempt to detect this and, if found, try to fix it by flipping SNPs on genes known to be coded in the reverse strand. After fixing, it is checked again to make sure the issue is no longer detected, otherwise the file is rejected. This check is important as unfiltered data of this kind would create a serious anomaly in the allele distribution and influence results of some analyses.

#### 4.2.3 Check sequencing method for VCF format files

The variant-call-format (VCF) format was designed for NGS data, as it presents whole genome data with good storage efficiency by only listing SNPs for which genotypes are non-identical to the reference genome. In the consumer dataset however, we observed that many VCF format files contain over 50% of their entries reported as identical to the reference genome. The reason for this turned out to be that they are the result of genotype array data being converted to an NGS VCF format. Another feature of these genotype-converted VCF files is that many entries lack information about the alternative allele. This is because it is not provided in the genotype data it was converted from. The program will automatically deduce and separate out genuine NGS files, and label those from the genotype-derived VCF files as genotyping results.

### 4.3 SNP sanity check

#### 4.3.1 Basic SNP check

Surprising entries can be seen when processing genotype data that is not directly obtained from a primary source. Entries that contain unexpected characters are excluded. SNPs are checked for valid chromosome IDs, and positions should be included in the reference genome. Genotype calls should be 1 or 2 digits, and correspond to valid amino acid or in/del characters. Positions with no calls, e.g. ‘-’, ‘_’, ‘0′ are also noted and excluded.

#### 4.3.2 Match the genotyping data to a cluster and flag those that match no cluster

The genotyping data are matched to one of the array clusters identified in Section 3.2. A cluster is assigned if it covers 80% of the SNPs in the genome. This is important because genotyping data are subjected to quality control of different lists of SNP positions depending on their cluster (see below, 4.3.3). Genomes that do not have similarity to any cluster are also labelled.

#### 4.3.3 Remove SNP positions in the array that are not statistically plausible

This is one of the most important and challenging parts of quality control for genotyping data. We have generated lists of SNPs in the public DTC datasets that do not seem credible when compared to an independent source of allele frequencies. Common practice for medical cohorts is to perform SNP quality control using PLINK to exclude the problematic positions before using the data for research [30], using statistics such as Hardy-Weinberg equilibrium P-value. In the absence of consistently generated cohort data, we make use of the cluster assigned in step 4.3.2 for quality control of the SNPs. Our approach is to use allele distributions from the 2504 genomes in the 1000 genomes project (1000G) as a reference, and for each cluster generate a list of SNPs that fail a Z-score based comparison test. Since the ethnic distribution of open genomes has an over-representation of individuals of European descent relative to that of the 1000G, we require a Z-score failure on both the entire 1000G and separately on the European subset of 1000G. Based on the assigned cluster from 4.3.2, the corresponding list of genotype positions is excluded from an individual genome.

#### 4.3.4 Check for genome identity and close familial relationship

As an additional check, the final step is to compare genetic similarity between genome files to detect and report files which correspond to the same individual (or identical twin), and files which correspond to immediate relatives (siblings or parents/children). For some research questions it may be desirable to filter out duplicates or related genomes, so the similarity is calculated as a percentage overlap of positions reported in both files. Cutoffs for duplicates and close relatives are derived from the distribution observed in Fig. 5. This feature is included in the package, but not available on the web service, which is for single genomes only.

### 4.4 Results of quality control on test genomes

After the pipeline, 6477 DNA data were identified as genotyping results and a typical microarray type is identified for 99% of them (Fig. 3). A single combined data-freeze for the 5393 OpenSNP genotyping data whose microarray type was identified by pipeline is available for download from https://supfam.mrc-lmb.cam.ac.uk/GenomePrep/.

**Fig. 4:**
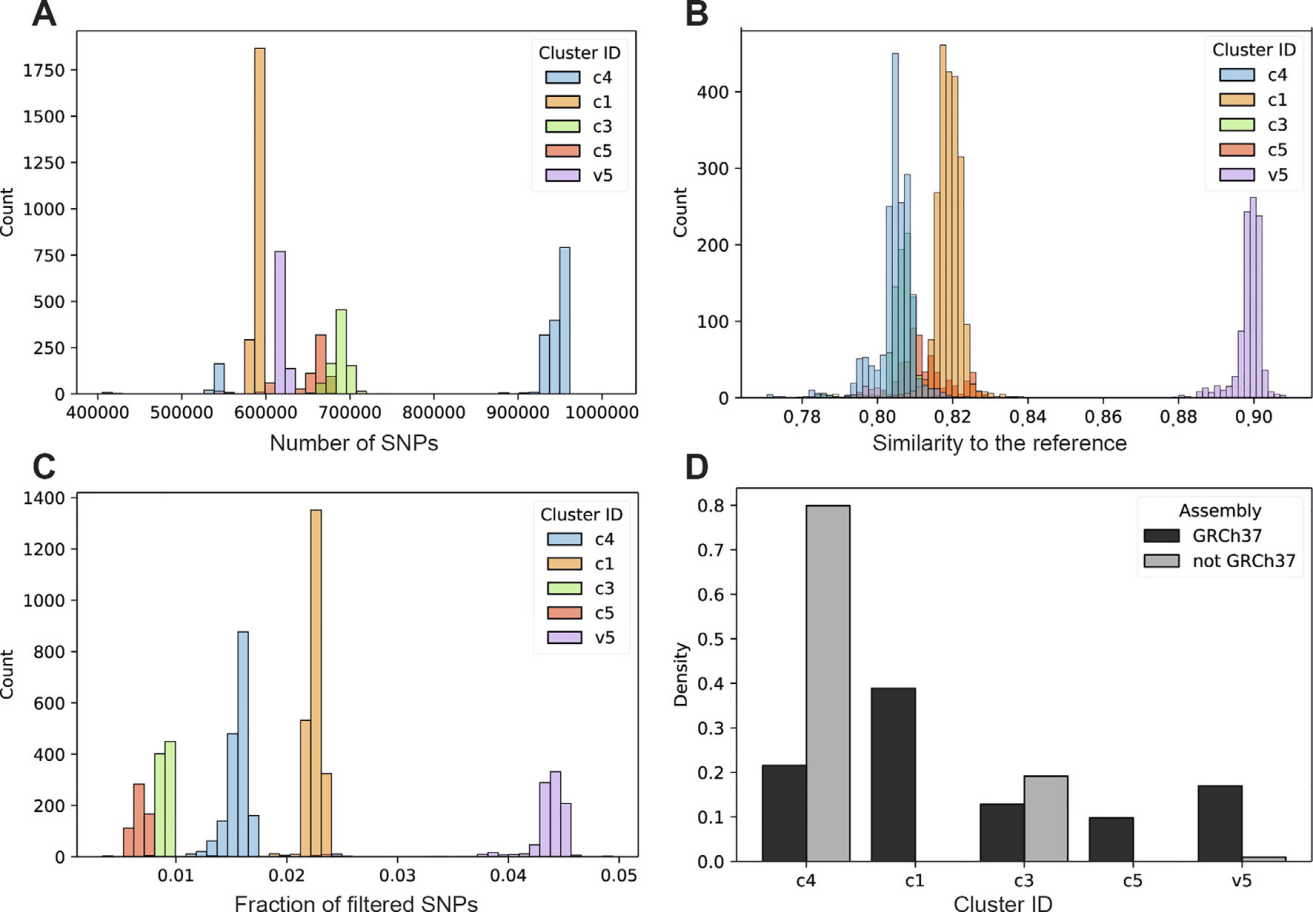
Analysis split into the genotype data clusters identified in Fig. 2 and Table 2C. Distribution of (A) total number of filtered SNPs, (B) similarity to the reference genome, and (C) fraction of excluded SNPs for each cluster. (D) Distribution of number of genomes in each cluster by the genome reference assembly.

It is worth noting that the new chip Array cluster v5 (Illumina GSA) is found to be on average the most similar to the reference (Fig. 4B), and it also contains the most fraction of questionable SNPs (Fig. 4C). We also observed that the GRCh36 genomes tend to fall in clusters c3 and c4 (Fig. 4D), which correspond to v1 of AncestryDNA and the v3 of 23andMe 1 million array respectively. This observation corroborates our previous deduction of array chips shown in Table 2, as the c1 (23andme v4), c5 (AncestryDNA v2) and v5 (Illumina GSA) all appeared later in the time line. A small number of GRCh38 genomes were seen in the v5 cluster, which is currently the most popular chip array.

**Fig. 5:**
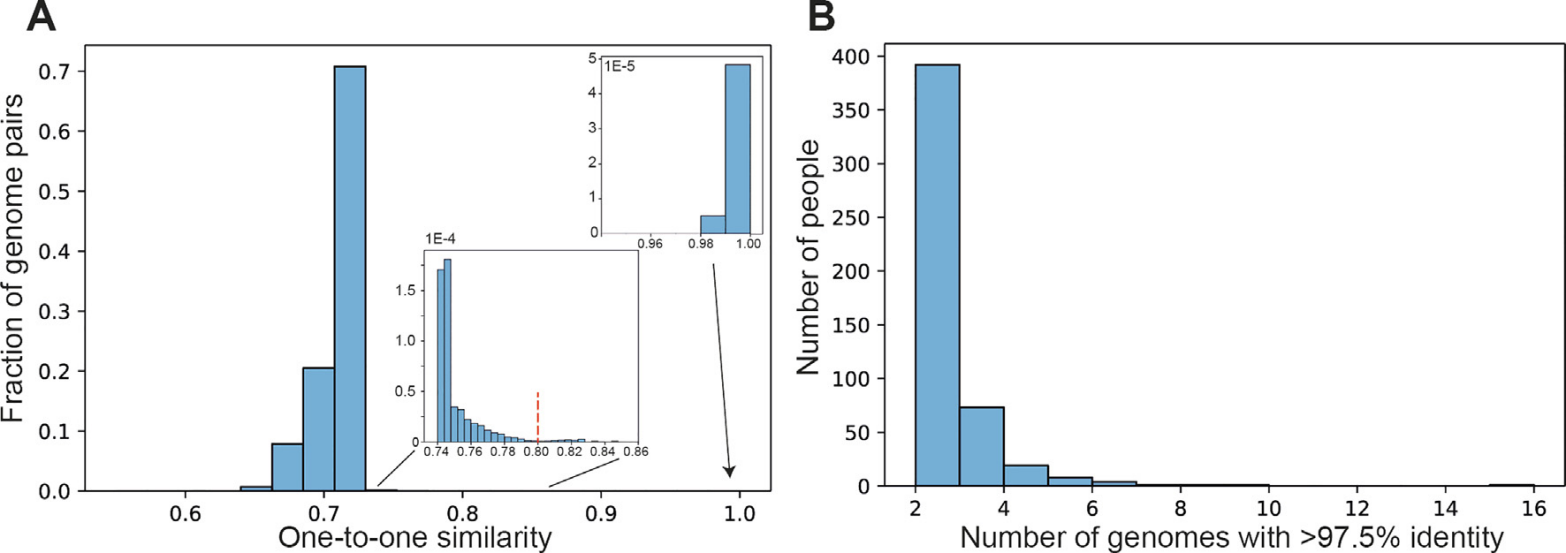
Pairwise similarity comparison of 6477 genotype files. (A) Distribution of one-to-one similarities between genotype data. Insets: above, showing 1070 pairs with greater than 97.5% identity but not a single pair between 90% and 97.5%, indicating a clear binary cutoff for genome files that originate from the same person (or identical twin); below: showing cut-off area (80%) used for finding close family relatives. (B) Number of people who have more than one highly similar open genomes, they can be identical twins, data from different sequencing sources, or uploaded more than once to different platforms.

The results of pair-wise comparisons between all genomes enable the identification of genomes from the same genetic origin, and close relatives (Fig. 5). 500 people (or identical twins) were found to have uploaded more than one open genome. An estimated 5781 different genetic origins were found within the entire cohort of open genomes.

## 5 Discussion

Consumer genome sequencing products are developed not only by technological advancement, but also under the economics of market pressure to be financially beneficial to the company. We have observed companies developing different genotyping arrays for different purposes (Table 1), and the differences create a significant barrier to cross-provider analyses. In the future, even as we ultimately see NGS sequencing take over for DTC customers as it gets cheaper, we will likely see this issue persist as DTC genomes emerge with various sequencing depth from different sequencing technologies and bioinformatics pipelines. This presents a current and future challenge for widespread use of DTC genomes for research. As consumer genomes continue to grow and outstrip medical cohorts in size, cross-provider tools such as *GenomePrep* made available through this work, will be required to capitalise on the massive potential of this body of data as a research resource.

Most genetic analysis requires a cohort of data to establish variant statistics. So what one consumer can do with the raw genome data is very limited. The power of consumer genomes comes when there is a large amount of data, which is currently accessible as a homogeneous dataset by only the companies that initially conducted the sequencing. Increasingly however, as these companies move to monetise their access to this resource, savvy consumers who control their own data will look outside the original provider for ways to share and use their genome. We will see power move away from providers as large scale online match-making services between consumers and e.g. drug companies emerge.

We were only able to develop this quality control pipeline, thanks to the thousands of people who donated their data to open research [31], [32]. At minimum, hundreds of sequencing results are needed to establish the statistical power necessary to determine alleles that are likely errors in each cluster of genotypes. Genomics and human genetics are scientifically fundamental and commercially valuable [33]. It is our hope and expectation that, whatever happens in the commercial marketplace, there will continue to be an abundance of volunteers stepping forward to support academic medical (and other) research by contributing their genome. At present it seems likely that individuals will ultimately have major control over how, and by whom their own genome is used.

Consumer genetic data is going to keep growing. At the moment consumer data used for research is via consumers opting into research consent, e.g. 23andMe, AncestryDNA; sometimes this data, or access to customers is sold as a package to commercial and academic parties. Academically, genomic data is generated by institutions who usually recruit participants with particular phenotypes and where sequencing is supported by research grants. At some point, as most people have control of some form of personal sequence data, these two main sources of genome sequencing will cease to dominate. Already some academic studies allow participants to download the DNA file, e.g. the genes-for-good project [18]. The new generation of DTC sequencing companies are offering more incentives to customers, such as profit-sharing the monetisation of data with the customer, while others such as LunaDNA, offer company shares in exchange for genetic and phenotypic data [34].

In short, the number of people with their genome sequenced who are willing to participate in research will grow, and they are likely to come from diverse sources and from various methods. Open-source tools currently available to interpret these data are mostly focused on parsing genomes from various sources and not yet offering quality control, e.g. snps [35]. The *GenomePrep* quality control pipeline, developed on the goodwill of open genome data, addresses the problem in the context of the present: genotype arrays. The tool is available as a web service at http://supfam.mrc-lmb.cam.ac.uk/GenomePrep/ and as an open source Git repository at https://github.com/changlubio/GenomePrep/.

## Funding

This work was supported by the Medical Research Council, as part of United Kingdom Research and Innovation (also known as UK Research and Innovation) [MC_UP_1201/14].

## CRediT authorship contribution statement

**Chang Lu:** Conceptualization, Investigation, Methodology, Software, Writing - original draft, Data curation, Visualization. **Bastian Greshake Tzovaras:** Data curation. **Julian Gough:** Conceptualization, Resources, Investigation, Writing - review & editing, Funding acquisition.

## Declaration of Competing Interest

The authors declare that they have no known competing financial interests or personal relationships that could have appeared to influence the work reported in this paper.
